# Understanding plant responses to stress conditions: redox-based strategies

**DOI:** 10.1093/jxb/erab324

**Published:** 2021-08-11

**Authors:** Francisco Javier Cejudo, Luisa M Sandalio, Frank Van Breusegem

**Affiliations:** 1 Instituto de Bioquímica Vegetal y Fotosíntesis, Universidad de Sevilla, and Consejo Superior de Investigaciones Científicas, 41092 Sevilla, Spain; 2 Department of Biochemistry, Cell and Molecular Biology of Plants, Estación Experimental del Zaidín, Consejo Superior de Investigaciones Científicas, 18008 Granada, Spain; 3 Department of Plant Biotechnology and Bioinformatics, Ghent University, 9052 Ghent, Belgium; 4 Center for Plant Systems Biology, VIB, 9052 Ghent, Belgium

**Keywords:** Cysteine, oxidative stress, plant acclimation, reactive oxygen species, reactive nitrogen species, reactive sulfur species, redox regulation

**Plant acclimation to environmental conditions involves multiple interactions between hormones and other signaling molecules. A lot of attention has been devoted to the signaling function of reactive oxygen species and their relationships to thiol-dependent redox regulations. Recently, new developments in proteomic techniques have revealed the relevant signaling effect of reactive nitrogen species and reactive sulfur species. Together, they cause post-translational modifications in proteins that participate in signaling networks, such as those responding to hormones, allowing the rapid response of plants to environmental cues.**

One of the consequences of climate change is an increase in CO_2_ emissions, drought, flooding, and soil salinization, whereas another outcome of anthropogenic actions is the accumulation of heavy metals that are very harmful to all types of organisms. As these processes will reduce plant production and product quality, it is necessary to deepen our understanding of the mechanisms that regulate plant responses to stress conditions and contaminants, as well to improve plant and food production under these unfavorable conditions by developing and selecting more stress-tolerant species. The different stages of a plant’s life cycle are tightly adapted to the environment. Thus, environmental cues, including temperature, photoperiod length, and light quality and intensity, among others, exert a great influence on seed germination, vegetative growth, and differentiation of reproductive organs. However, as sessile organisms, plants must withstand the unpredictable changes in their surroundings with a possibly adverse impact on plant growth and, hence, crop productivity. Plant acclimation to stresses is based on sophisticated signaling networks, and the plant growth-controlling hormones participate in the rapid response of plants to environmental conditions. Biotic and abiotic stresses that affect oxygen, nitrogen, and sulfur metabolism trigger the production of reactive oxygen (ROS), nitrogen (RNS), and sulfur (RSS) species. As these species are highly reactive with proteins, nucleic acids, and lipids, they might be harmful when they accumulate at high levels. Nevertheless, they also have an important signaling function, based on their ability to produce post-translational modifications (PTMs) in proteins. Among the different amino acids, such as methionine, tyrosine, lysine, and arginine, that are targets of these species, the most important one is cysteine (Cys) because of the high reactivity of the thiol group (Zaffagnini *et al.*, 2019). The dithiol–disulfide exchange of Cys residues in redox-sensitive proteins provides the framework for redox regulation (Box 1). ROS, RNS, and RSS cause PTMs of Cys residues, hence interfering with the redox regulatory network of the cell. Therefore, these species are important signaling molecules that can rapidly trigger reversible PTMs in many proteins, provoking changes in the cell proteome and fine-tuning protein function, localization, stability, and interactions to mitigate the potential damage of environmental stresses. Therefore, PTMs are crucial for the detection of alterations in the surroundings and for the elicitation of specific plant responses.

**Box 1. Cysteine modifications affecting thiol-dependent redox regulation**

Thiol–disulfide exchange between well-conserved Cys residues (circled) is the basis of redox regulation, a universal regulatory mechanism that allows the rapid response of metabolic and signaling pathways to the environment. Reduction is catalyzed by the disulfide reductase activity of glutaredoxins (GRXs) and, to a greater extent, thioredoxins (TRXs) that use reducing equivalents from NADPH and from reduced ferredoxin in chloroplasts. The oxidation mechanism is less well understood, although H_2_O_2_ has recently been proposed to act as the final electron sink in the chloroplast enzyme oxidation process in the dark that is mediated by TRXs and peroxiredoxins (PRXs) (Cejudo *et al.*, 2021). Environmental conditions trigger the increase of ROS, RNS, and RSS that react with Cys thiols, hence altering this redox-regulatory network. H_2_O_2_ causes the oxidation of Cys thiol to sulfenic (SOH), sulfinic (SO_2_H), and sulfonic (SO_3_H) acids. Both SO_2_H and SO_3_H are considered irreversible modifications, except that in PRXs the SO_2_H form of peroxidatic Cys is reversed to thiol by sulfiredoxin. Cys thiol may also be oxidized to SO_2_H by the plant cysteine oxidase (PCO). Additionally, Cys thiols may become *S*-nitrosylated by NO and derivatives (RNS), *S*-pursulfidated by H_2_S, and glutathionylated.

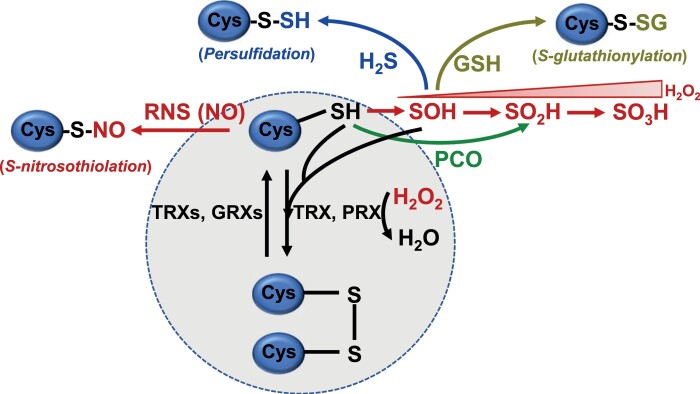

## Cysteine PTMs triggered by ROS, RNS, and RSS

Aerobic metabolism produces hydrogen peroxide (H_2_O_2_), superoxide anion (O_2_^–^), singlet oxygen (^1^O_2_), and hydroxyl radicals (OH·), which are the most important ROS in plant cells (Waszczak *et al.*, 2018). H_2_O_2_ is highly reactive toward Cys thiols, with their oxidation to sulfenic (SOH), sulfinic (SO_2_H), and sulfonic (SO_3_H) acids as a consequence (Box 1). These redox-dependent modifications interfere with the regulatory mechanism of the proteins by affecting their activity, stability, and/or cell location. Moreover, H_2_O_2_ exhibits a high permeability through biological membranes, allowing the transmission of its signaling activity between cells and to different plant parts (Mittler, 2017). The most important RNS is nitric oxide (NO), the synthesis of which is still not fully understood, but it has been shown to be produced either as a side reaction of nitrate reductase in the cytosol, or by electron transfer to nitrite in mitochondria, although an NO-like activity with arginine has also been described in plants (Astier *et al.*, 2018). NO is a relatively unreactive molecule, with a preference for other radicals and metals, modifying Cys residues by *S*-nitrosylation (Box 1) (Zaffagnini *et al.*, 2019). PTMs that depend on ROS and NO can interact with one another, playing both a synergistic and an antagonistic role depending on the proteins involved (Romero-Puertas and Sandalio, 2016). Finally, the most relevant RSS, hydrogen sulfide (H_2_S), is produced from Cys in plastids in a reaction catalyzed by *O*-acetylserine thiol lyase (OASTL), through l-Cys desulfhydrase in the cytosol and β-cyanoalanine synthase in the mitochondria (Box 1) (Aroca *et al.*, 2018).

## The signaling function of ROS in plant development and responses to stresses

The essential function of ROS in plant developmental processes is widely recognized (Mhamdi and Van Breusegem, 2018). In this special issue, Considine and Foyer (2021) discuss the molecular mechanisms of the ROS signaling function as cell fate determinants, emphasizing the synergistic and antagonistic ROS interactions with the plant hormones abscisic acid (ABA) and ethylene that mediate stress-induced signals affecting plant development. Virtually any type of biotic or abiotic stress provokes the imbalance of ROS production and scavenging, hence triggering ROS accumulation. To fully understand the ROS signaling function, an important issue to be considered is their production origin and spatial distribution. ROS are generated in chloroplasts, mitochondria, and peroxisomes as a consequence of electron transport chains and metabolic pathways that occur in these organelles. In the apoplast, ROS are mainly produced by NADPH oxidases and peroxidases embedded in the plasma membrane, and their production is initiated in response to stress. ROS from different organellar sources trigger specific signaling pathways that hint at the complex metabolic and signaling activity of ROS in plant cells (Phua *et al.*, 2021). A ROS-affected signaling pathway that involves different cell compartments is the chloroplast to nucleus retrograde signaling that communicates the chloroplast state to the nucleus to coordinate the expression of nuclear genes encoding chloroplast-localized proteins (Phua *et al.*, 2021). Plants, like other organisms, can anticipate cyclic environmental conditions by the circadian clock, which is essential for acclimation to biotic and abiotic stresses. In addition to the genetic central oscillator based on transcriptional and translational feedback loops, 2-Cys peroxiredoxin (Prx), a H_2_O_2_-scavenging enzyme, has been proposed to act as a universal marker of metabolic circadian rhythms (Edgar *et al.*, 2012). These findings suggest a tight relationship between ROS and the circadian clock, although the molecular mechanisms are poorly understood. A search for daytime-regulating genes that encode enzymes generating and scavenging H_2_O_2_ under different stress conditions allowed the establishment of ROS level timing throughout the day–night cycle. These observations support the notion that plants have developed mechanisms integrating information from external stimuli with the time of day to adjust the gene expression and orchestrate the response to environmental stresses (Jiménez *et al.*, 2021).

## Interplay of ROS, RNS, and RSS signaling activity in plant response to stress

The present global warming and its severe impact on plant growth and productivity have attracted increasing interest on the effects of temperature on plant growth. The finding that mutant plants with altered nitrosothiol (SNO) and NO levels exhibit thermotolerance defects has led to the identification of the RNS signaling function in plant tolerance to high temperature, as discussed by Sánchez-Vicente and Lorenzo (2021). The PTMs triggered by RNS comprise Cys *S*-nitrosation, metal *S*-nitrosylation, and nitration of tyrosine residues, and affect plant responses to temperature by modulating different signal transduction pathways that activate oxidative stress defense, accumulation of osmolytes and heat shock proteins (HSPs), and protection of the photosynthetic machinery. Moreover, large-scale proteomic analyses have revealed the participation of RNS in plant responses to low temperatures, as implied by the modifications of the plant nitroproteome in response to cold stress. Sánchez-Vicente and Lorenzo (2021) summarize the evidence that supports the crucial role of NO in maintenance of cell membrane stability, increase in antioxidant defense, and PSII recovery in response to low temperature treatments.

Other effects of global warming are heavy rainfalls, with transient hypoxia to plants in flooded lands as a consequence, thereby leading to severe agronomic costs and yield losses. Thus, the mechanisms by which plant cells sense oxygen, and the physiological and molecular adaptations that occur when plants under hypoxia become reoxygenated (the hypoxia to normoxia transition) have received a lot of attention. In this issue, León *et al.* (2021) describe the role of the group VII transcription factors of the ethylene response factor (ERFVII) family in plant oxygen sensing. The mechanism involves oxidation of the Cys2 residue from thiol to sulfinic acid at the N-terminal side of the protein in a reaction catalyzed by the plant cysteine oxidase (PCO) (Box 1) that enables the degradation of the transcription factor. Thus, the factor remains stable under hypoxia and becomes degraded after reoxygenation. Interestingly, PCO-encoding genes are up-regulated by NO, hinting at the participation of RNS in oxygen sensing in plants, although the mechanism remains unknown. In addition, the reoxygenation triggers deep changes at the metabolomic, transcriptomic, and epigenetic levels (León *et al.*, 2021).

Most studies of plant stress responses focus on a single stress condition. While these approaches are useful for dissecting signal transduction pathways that modulate the plant response to the stress, these experimental designs are far from reflecting the achievement of the actual plant that must react to multiple stresses in nature. In other words, plant acclimation results from the complex interplay between different signaling pathways, a still poorly deciphered issue. The identification of the signaling pathways common to different stresses might possibly be achieved by taking advantage of the large amount of metabolomic, transcriptomic, and proteomic data publicly available. As a step in this direction, Romero-Puertas *et al.* (2021) used transcriptomic analyses in search of Gene Ontology categories common to abiotic cadmium stress and biotic (fungal treatment) stresses in Arabidopsis (*Arabidopsis thaliana*) and rice (*Oryza sativa*). This study identified redox signaling at the crossroads of both stresses, emphasizing the central role of glutathione metabolism, in particular of the glutathione *S*-transferase (GST) genes. Additional genes important in the response of plants to both stresses are genes that encode HSPs associated with endoplasmic reticulum (ER)-associated degradation (ERAD) and genes involved in chitin response (Romero-Puertas *et al.*, 2021).

Of great interest for the analysis of the interplay between ROS, RNS, and RSS are legume nodules. Nodules generate H_2_O_2_ by plasma membrane-localized NADPH oxidase and NO by bacterial denitrification, nitrate reduction, and the mitochondrial electron transport chain in the hypoxic tissue, as well as H_2_S by plant and bacterial enzymes (Matamoros and Becana, 2021). An analysis of proteomic data identified different PTMs (e.g. Met sulfoxidation, Cys sulfenylation, *S*-nitrosylation, *S*-glutathionylation, *S*-persulfidation, Tyr nitration, carbonylation, and glycation) in many nodule proteins. These findings reveal the importance of ROS, RNS, and RSS in the alteration of the nodule redox homeostasis and the modulation of the abiotic stress response (Matamoros and Becana, 2021).

Finally, the signaling function of H_2_S in the plant’s response to stress is gaining increasing relevance. Interestingly, H_2_S is unable to react directly with Cys thiols, but reacts with the sulfenic form (Box 1), implying that H_2_O_2_-dependent sulfenylation is required for persulfidation and, actually, a concurrence of up to 82% exists between the sulfenylome and persulfidome in plants (Aroca *et al.*, 2021). Persulfidation modulates plant responses to environmental cues by affecting different signal transduction pathways. It is notewhorthy that the persulfidation of key components of the ABA signaling network has uncovered that this PTM plays an central role in the control of stomatal aperture, constituting an excellent example of the interplay between RSS and hormone signaling (Aroca *et al.*, 2021).

The possibility to improve crop productivity under adverse environmental conditions is an anticipated result from the increasing knowledge on the signaling functions of ROS, RNS, and RSS, and on their relationship with the hormonal regulation of plant development and stress responses. Although, as mentioned above, any cell compartment contributes to these signaling networks, the chloroplasts play a very prominent role. Chloroplasts are the source of metabolic intermediates that support plant growth and, hence, plant development. Moreover, chloroplasts have an important signaling function as a major source of ROS, RNS, and RSS, as well as of plant hormones and hormone precursors. However, stressful environmental conditions may damage the chloroplasts that could potentially be harmful given the phototoxicity of many chloroplastic components. The complex mechanisms of chloroplast dismantling triggered in response to stress and the redistribution of metabolic resources to sink tissues during leaf senescence are described by Domínguez and Cejudo (2021). Furthermore, the chloroplasts emerge as an important target in biotechnological strategies. Most of these approaches manipulate the redox networks of the organelle with the aim of improving the defense against environmental stresses that are aggravated in the present context of global warming, as discussed by Lodeyro *et al.* (2021).

## Conclusions and future perspectives

Due to their sessile nature, plants must be subjected to multiple adverse environmental conditions that affect growth and limit agronomic productivity, a state that is worsening in the present situation of global warming. Plants have evolved complex signaling networks to monitor environmental cues and acclimate their growth and development accordingly, a process in which plant hormones play an important role. The permanent improvement in proteomic methodologies that allow a more precise identification of protein PTMs will help to uncover the relevant signaling functions of ROS, RNS, and RSS in plant responses to environmental cues. These species trigger PTMs in many proteins, most frequently targeting their Cys residues, interfering with redox regulatory networks that participate in numerous signaling pathways, and fine-tuning plant growth and development to fluctuating environmental cues. A major challenge in this field is to gain insight into the interplay between different transduction pathways and the plant responses to combined stresses. This knowledge will allow the discovery of biotechnological strategies to improve plant productivity under increasingly adverse environmental conditions that are currently exacerbated by global warming.
